# Undergraduate musculoskeletal ultrasound training based on current national guidelines—a prospective controlled study on transferability

**DOI:** 10.1186/s12909-024-06203-6

**Published:** 2024-10-23

**Authors:** Andreas Weimer, Florian Recker, Thomas Vieth, Holger Buggenhagen, Christian Schamberger, Rainer Berthold, Svenja Berthold, Stephan Stein, Gerhard Schmidmaier, Roman Kloeckner, Ricarda Neubauer, Lukas Müller, Julia Weinmann-Menke, Johannes Weimer

**Affiliations:** 1 Clinic for Trauma and Reconstructive Surgery, University Clinic Heidelberg, 69118 Heidelberg, Germany; 2 Department of Obstetrics and Prenatal Medicine, University Hospital Bonn, Bonn, Germany; 3 Rudolf Frey Learning Clinic, University Medical Centreof the, Johannes Gutenberg University Mainz , 55131 Mainz, Germany; 4 Orthopedic practice, Wetzlar, 35578 Germany; 5 Department for Orthopaedics and Trauma Surgery, University Medical Centre Mannheim, 68167 Mannheim, Germany; 6 Institute of Interventional Radiology, University Hospital Schleswig-Holstein—Campus Lübeck, Lübeck, 23538 Germany; 7 Department of Diagnostic and Interventional Radiology, Mainz University Hospital, Mainz, Germany; 8 Department of Internal Medicine I, University Medical Center of the, Johannes Gutenberg Universitätsmedizin Mainz, Langenbeckstraße 1, Mainz, 55131 Germany

**Keywords:** Ultrasonography, Musculoskeletal ultrasound, Education, Medical, Undergraduate, Blended learning, Competency-based education, DOPS

## Abstract

**Introduction:**

Musculoskeletal ultrasound (MSUS) is integral to routine clinical diagnostics for musculoskeletal and joint disorders. This study aims to establish and validate a sonography course tailored to undergraduate medical students acquiring MSUS-specific skills at a German university.

**Methods:**

A blended learning training concept, comprising 24 instruction sessions of 45 min each, was designed based on the current national guidelines of the German Society for Ultrasound in Medicine (DEGUM). This program was integrated into the clinical phase of the undergraduate students’ medical education. The self-perceived improvement in competency and the effectiveness of the course design were evaluated using a a 7-point Likert scale questionnaire. Objective learning success was evaluated via a written test and a “Direct Observation of Practical Skills” practical exam. Control groups included medical students without MSUS training (control group 1) and doctors who had completed DEGUM-certified basic MSUS courses (control group 2). Both control groups completed the written test, while control group 2 also took the practical final exam. The study involved 146 participants: 56 were allocated to the study group, 44 to control group 1, and 46 to control group 2.

**Results:**

The study group rated their skills significantly higher after the course (*p* < 0.01). Participants expressed high satisfaction with the course design, the teaching materials, and the teachers. The study group's performance on the final written test was comparable to those of control group 2 (*p* = 0.06) and significantly superior to control group 1 (*p* < 0.001). Additionally, the study group’s performance on the practical final exam was not significantly different from control group 2 (*p* = 0.28), with both groups achieving scores exceeding 80%.

**Conclusion:**

Both subjective and objective measures of learning suggest that an MSUS course designed for postgraduates can be effectively adapted for undergraduate medical students. Incorporating MSUS training into the clinical curriculum is recommended to enhance future medical professionals' educational experience and practical skills.

**Supplementary Information:**

The online version contains supplementary material available at 10.1186/s12909-024-06203-6.

## Introduction

### Background

Sonography is becoming increasingly important in clinical patient care and is now a well-established diagnostic tool across almost all medical disciplines [1]. Additionally, sonography plays a crucial role in visualizing the musculoskeletal system, complementing other imaging techniques for detecting pathologies, making differential diagnoses, and supporting therapeutic interventions [2–5]. Therefore, it is essential that medical professionals receive early, comprehensive, and well-founded training in this dynamic examination method.

To meet this need, more ultrasound-specific training concepts are being integrated into both preclinical and clinical training for medical students [6–12]. Such programs often include a combination of peer-supported elective and compulsory courses, spanning a semester or in more compact formats [7, 13–15]. Some universities have developed and successfully implemented ultrasound curricula spanning several semesters [14–16]. This is often done through blended learning, a popular and effective method of instruction [17–20]. International professional societies advocate for an early start to training in ultrasound within medical degree programs and provide general recommendations about teaching staff numbers and expertise, curriculum design, teaching methods, teaching materials, interactivity, motivation, and resource management/facility [21–23].

## Research question & aim

Recommendations and guidelines from national and international professional societies such as the European League Against Rheumatism (EULAR), the Pan-American League of Associations for Rheumatology (PANLAR), and the German Society for Ultrasound in Medicine (DEGUM) [24–26] predominantly address specialist training for postgraduates and lack any specific requirements regarding course format, content or test instruments for students [27, 28].

Most evaluated training concepts focus on training for medical doctors [27, 29–33], with limited attention to undergraduate education [14, 27, 34–42]. Various teaching methods such as peer teaching, team-based learning, blended learning, mnemonic and metaphorical videos,and Peyton's Four-Step Approach have been employed to develop theoretical and practical skills [27, 28, 35, 38, 39, 42–46].

Several factors make musculoskeletal ultrasound (MSUS) particularly suitable for incorporation into student education:

1. Most superficial structures of the musculoskeletal system are easily visualized in sonography [47].
2. Dynamic visualization of the musculoskeletal system complements physical examinations and training enhances the examiner's understanding of functional anatomy [36, 48–50].
3. The non-invasive and radiation-free nature of sonography allows for repeated training and peer-to-peer instruction with minimal safety precautions [35, 43].
4. Pre-existing knowledge of sonography or other imaging procedures can induce steep learning curves when implementing the technique in clinical practice [30, 45].
5. Sonography can also be used in a point-of-care setting to focus on specific issues [3, 4, 51].

In addition, the updated version of the National Competency-Based Learning Objectives Catalogue of Medicine (NKLM Version 2.0) includes advanced clinical skills such as sonography of the musculoskeletal system, indicating these should be integrated into medical degree programmes in Germany [52].

This prospective study evaluates the extent to which an established, certified basic course concept from DEGUM, intended for medical doctors [24], can be adapted for undergraduate education. The focus is on developing theoretical and practical competencies, while outcomes will be compared with those of postgraduate medical doctors and untrained users. Furthermore, the study evaluates learners’ satisfaction with the training concept’s teaching methods, teaching materials, examination formats and tutors [24].

## Material and methods

### Study design and participants

**Fig. 1:**
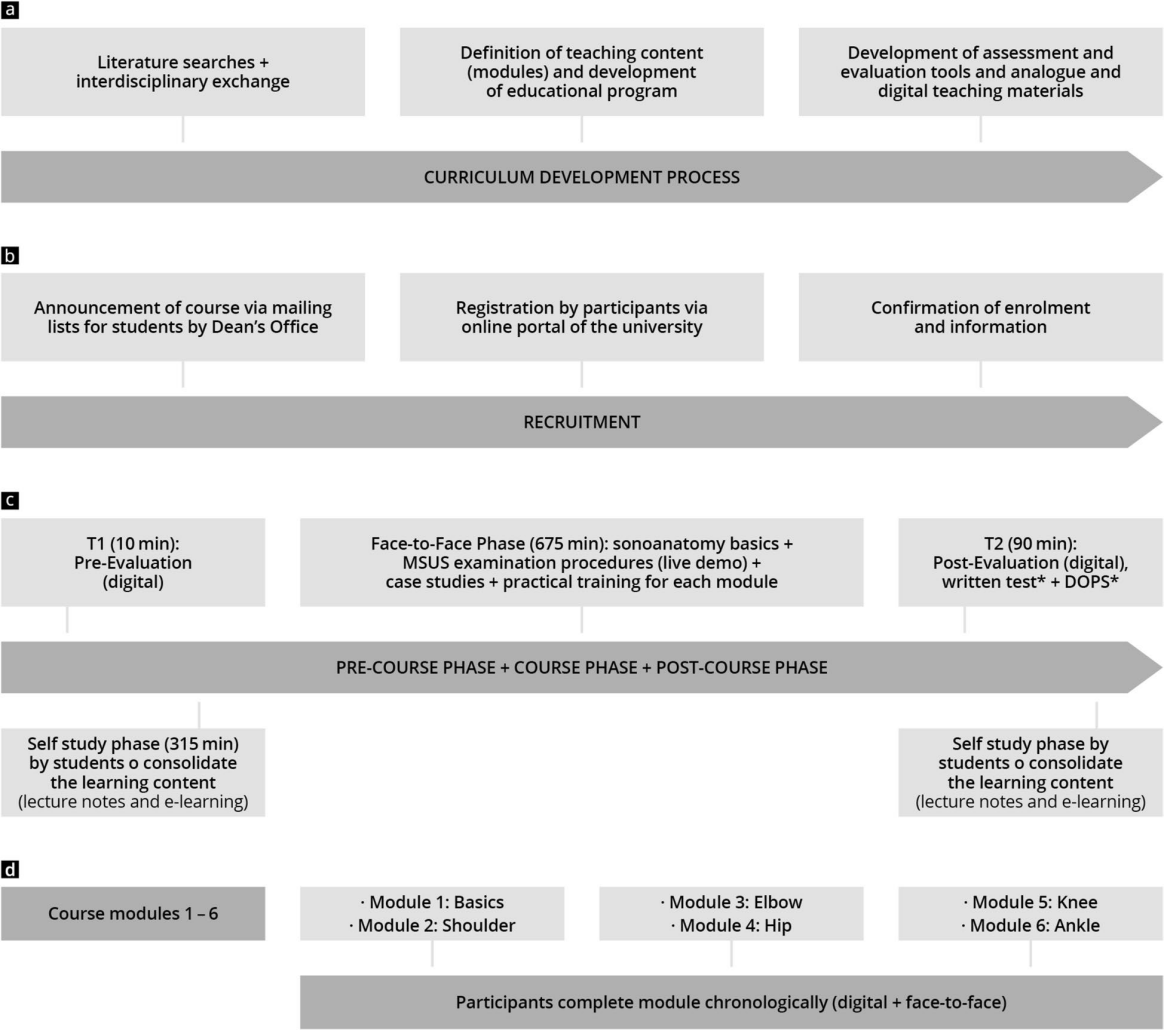
Study planning, study process and training concept; DOPS = Direct Observation of Procedural Skills Test. **a** MSUS curriculum development process; **b** Timeline of participant recruitment; **c** Course design including assessments; **d** Module sequence; *These tests were performed by the control groups as well as the study group

This controlled prospective study was conducted at a German university from 2023–2024 [53]. Digital evaluations were conducted at two points in time (T1 and T2), along with a theoretical examination and a Direct Observation of Procedural Skills Test (DOPS). These assessments measured objective competencies at the end of the training (primary endpoint) as well as the subjective increase in competency and satisfaction with the training curriculum (secondary endpoints) compared to two control groups [28, 54]. Figure 1 depicts the study design and the adapted contents from the DEGUM-MSUS postgraduate training curriculum [24].

Students from the fifth semester onwards could participate by voluntarily registering through the university's online portal after completing the pre-clinical part of the degree programme. All students in the clinical semesters received information about the course and how to register via e-mail from the study office. The inclusion criteria in the study analysis included complete course participation, completion of the evaluation and examination forms, and consent to participate in the study. By deciding for or against participation, the students did not receive any advantages or disadvantages in courses of the compulsory curriculum.

Control groups consisted of students without MSUS training (control group 1) and medical doctors in DEGUM-certified MSUS basic courses (control group 2) [24, 33]. Both control groups also completed the written test and a questionnaire about “baseline characteristics”, while control group 2 also completed the DOPS tests.

### Course design, learning objectives and teachers

The course was adapted from the DEGUM basics course [24, 47], current publications of (international) training concepts in university ultrasound teaching [27, 45, 55], and the specific learning objectives of the National Competency-Based Learning Objectives Catalogue for Medicine (NKLM) [56]. The German Association of Medical Faculties developed the NKLM emphasizing essential competencies, particularly practical skills and knowledge for medical students. The DEGUM Basic Course on the Musculoskeletal System, offered by the German Society for Ultrasound in Medicine, teaches ultrasound techniques and skills essential for assessing muscles, tendons, joints, and ligaments. This hands-on course targets healthcare professionals aiming to improve their diagnostic abilities in musculoskeletal disorders.

Blended learning was used as the teaching concept [17] and thus the training was segmented into a pre-course period, a face-to-face course, and a post-course phase, comprising a total of 24 sessions of 45 min each (see Fig. 1 and Supplement 1).

The theoretical and practical learning objectives are listed in Table 1. The course focused on teaching “basics of technique and equipment”, “anatomical understanding”, “sonomorphology of normal findings”, and carrying out “functional examinations”.

**Table 1:** Theoretical and practical learning objectives of the MSUS curriculum based on the basic MSUS DEGUM course [24, 47] and the NKLM [56]

Theoretical learning objectives	Practical learning objectives
• Ultrasound machine design including knobology • Transducer types • Technical and equipment basics • Modes (B-Mode and Color Doppler) • Basic anatomy of the musculoskeletal system • Understanding of ultrasound orientation views/sections • Steps in the examination of shoulder, elbow, hip, knee and ankle • Options for documenting findings • Recognition of pathologies	• Machine setup (transducer selection, preset selection, image optimization) • Mastering transducer handling (holding, movements, stabilization, connection) • Drawing ultrasound orientation views • Functional examination of joints in sonography • Documentation of findings recorded on the ultrasound machine

The speakers and tutors for the total of three student courses of 25-course places each were specialists (1x), residents (2x), and students in their clinical year rotation (2x). All speakers and tutors had already carried out more than 200 independent sonographic examinations of the musculoskeletal system and received additional training and a briefing in advance.

### Preparation/pre-course phase and study material

In this phase, the previous experience of participants and a self-assessment of skills were gathered through a digital questionnaire (T1). Following this, lecture notes (with an estimated processing time of approximately 180 min) and access credentials to an e-learning platform (with a processing time of 135 min) were provided before course preparation information was handed out.

### Course phase

During the face-to-face course phase, the participants alternated between theoretical impulse lectures with live demonstrations in the plenum (210 min in total) and practical training sessions (570 min in total) over two days. In practical training, small groups of five or six participants were supervised by one tutor.

A total of four ultrasound devices (two mid-range devices and two pocket devices), each equipped with high-frequency linear probes and special MSUS presets, were used for the practical exercises. After the practical exercises, participants completed a digital evaluation, a written test, and a practical exam of 90 min at T2.

### Post-course phase

The lecture notes and e-learning platform facilitated the long-term retention and memorization of the course content.

### Teaching materials

A combination of digital learning platforms (e-learning) and analogue lecture notes was used for the preparation and follow-up of the face-to-face course phase in a blended learning approach. The orientation sections of the respective joint region were listed in the lecture notes, including explanations and links to examination videos. The e-learning included an online video course (9 videos, approximately 15 min each) available on the online Moodle platform. Assessment tools used were questionnaires, a written test, and a practical examination.

Theoretical and practical competency levels were assessed through a written and practical exam at the end of the course (T2). The subjective acquisition of skills and satisfaction with the training curriculum were measured using digital evaluation forms before (T1) and at the end of the course (T2). The questionnaires addressed “personal characteristics,” “previous experience,” “attitude toward ultrasound teaching/future perspectives,” “curriculum evaluation,” “examination formats,” “teaching materials,” “tutors,” and “subjective assessment of competency” using various sub-items. Dichotomous questions, free text questions, and questions with a seven-point Likert answering format (1 = strongly agree; 7 = strongly disagree) were used.

The written test (out of a maximum of 72 points with a processing time of 30 min) assessed “Ultrasound basics” (23 points) and “Normal findings” (total 49 points) of the “Shoulder” (21 points), “Elbow” (6 points), “Hip” (6 points), “Knee” (max. 9 points) and “Ankle” (max. 7 points) by labelling of structures in standard cross-Sects. [47, 57]. See Supplement 2 for an example question.

The practical exam (out of a maximum of 49 points and 10 min per participant) was carried out as a DOPS [54, 58] on healthy volunteers (the participants examined each other). The different areas of competency were assessed according to a modified Objective Structured Assessment of Ultrasound Skills (OSAUS) scale [59]. The areas tested in the DOPS included “patient guidance/communication/indication” (max. 10 points), “transducer handling” (max. 8 points), “examination” (max. 8 points), “image description” (max. 4 points), “measurements/functional examination” (max. 6 points), “image documentation” (max. 1 point) and “interpretation of examination findings/statement of further action” (4 points). In total, each participant completed one of six possible DOPS scenarios (see Supplement 3 for an example sheet).

### Statistics

Data collection was carried out using the survey and test tool LimeSurvey (LimeSurvey GmbH, Germany), along with written questionnaires and practice exam sheets. All data were saved with Microsoft Excel (Version 16.0). Statistical analyses were performed in Rstudio (Rstudio Team [2020]. Rstudio: Integrated Development for R. Rstudio, PBC, http://www.rstudio.com, last accessed on 20 04 2024) with R 4.0.3 (A Language and Environment for Statistical Computing, R Foundation for Statistical Computing, http://www.R-project.org; last accessed on 20 04 2024). Where possible, a main scale score was made from the average of the subscale scores. The internal consistency of the evaluation scales was tested and ensured by calculating the reliability according to Cronbach’s alpha. Binary and categorical baseline variables are given as absolute numbers and percentages. Continuous data are given as median and interquartile range (IQR) or as mean and standard deviation (SD). Categorical variables were compared using chi squared test and continuous variables using the T-test or the Mann–Whitney U test. In addition, parametric (ANOVA) or non-parametric (Kruskall-Wallis) analyses of variance were calculated and further explored with pairwise post hoc tests (T-test or Mann–Whitney U). Finally, a multivariate linear regression model was produced to compare the influence of individual factors. *P*-values < 0.05 were considered statistically significant. A power analysis yielded a minimum sample size of 34 per group (study/control 1/control 2) for an effect size of 0.8, at a significance level of 0.05 and a desired power of 0.90.

## Results

### Data description

The reliability tests, as measured by Cronbach’s alpha, indicated that the internal consistency of the main scales ranged from 0.8–0.9 and did not vary considerably.

### Group characteristics

A total of 146 participants (PT) were included in the study (see Supplement 4). These included 56 participants in the study group, 44 participants in control group 1, and 46 participants in control group 2. The mean age of the study group (25 ± 4.0 years) was significantly lower (*p* < 0.001) than that of control group 1 (27 ± 3.3) and control group 2 (37 ± 8.7). Most participants in the study group (55%) and control group 2 (63%) were male. Within control group 2, most participants were in specialist training (59%) in the field of orthopaedics and trauma surgery (76%). Both the number of ultrasound examinations observed and those performed were significantly higher in control group 2 (*p* < 0.0001) than in the other two groups.

### Subjective evaluation

**Fig. 2:**
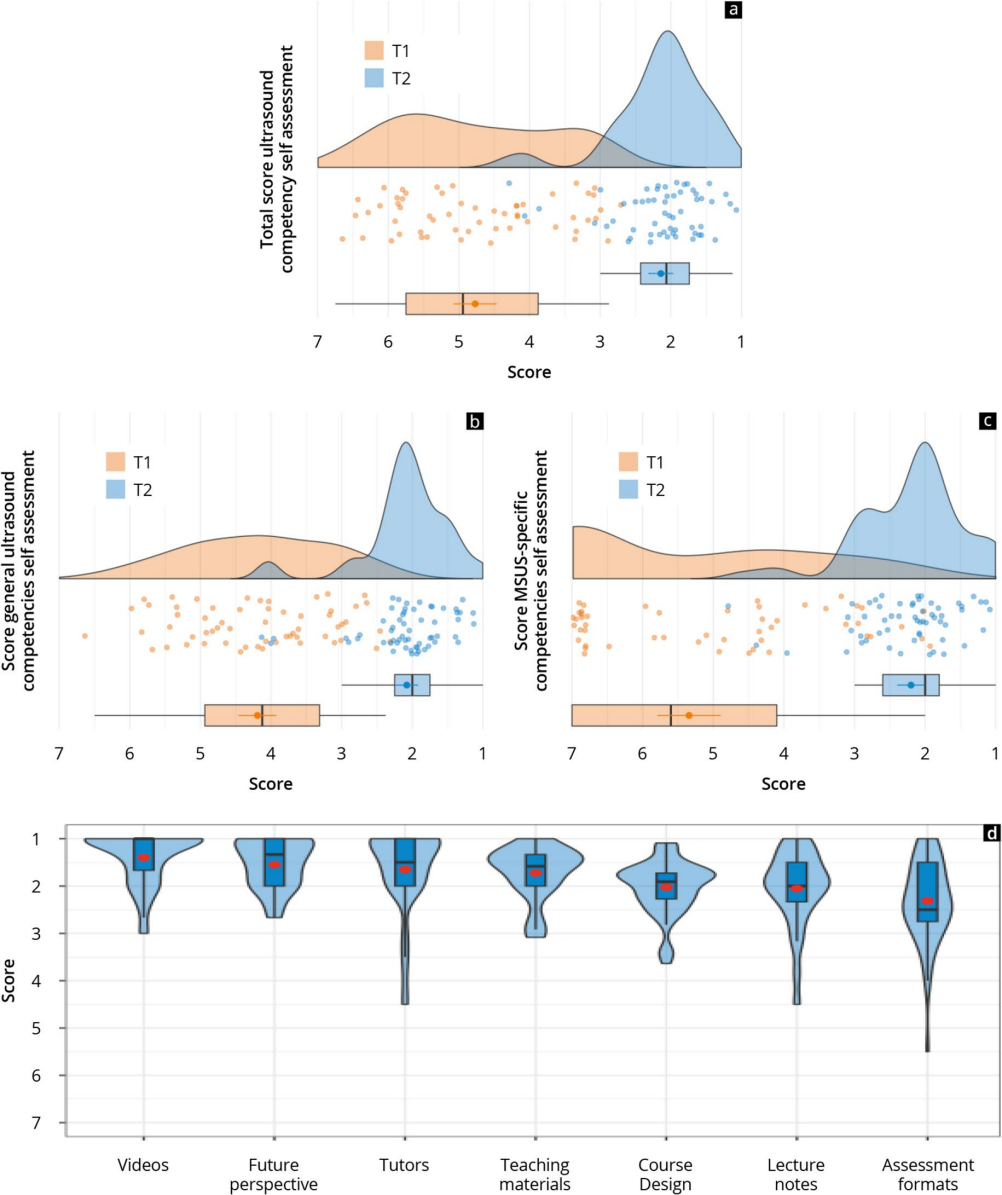
Results of the subjective competency assessment before (orange) and after (blue) completion of the curriculum regarding the “overall ultrasound competencies” (**a**), the “general ultrasound competencies” (**b**), and the “MSUS-specific competencies” (**c**), as well as the evaluation of the design (**d**). Likert answering formats with 1 = very good/completely and 7 = very bad/not at all. The visualisation was created using raincloud and violin plots

The participants’ subjective competency development is presented in Fig. 2 and Supplement 5. A significant increase in self-perceived competence was measured both in the overall score (Δ2.6 ± 1.4) and in the categories “general ultrasound skills” (Δ2.1 ± 0.9) and “MSUS-specific skills” (Δ3.1 ± 1.8) (*p* < 0.001). This also applied to all sub-items.

Table 2 and Fig. 2 show the evaluation results of the main and sub-items of the course design, specifically “future perspective”, “course concept”, “examination formats”, “teaching materials”, and “tutors”. All the main items queried except for the “examination formats” were evaluated in positive scale ranges on average (≤ 2.0 SP). “Future perspectives” (1.5 ± 0.5), “teaching materials” (1.7 ± 0.6) and “tutors” (1.7 ± 0.9) were rated particularly positively. The “videos” of the online module (1.4 ± 0.5) were evaluated significantly (*p* < 0.01) better than the “lecture notes” (2.1 ± 0.8). As part of the main item course concept (2.0 ± 0.6), the evaluation of the “course design” (1.6 ± 0.8) and the “relationship between theory and practice” (1.5 ± 0.7) were significantly (*p* < 0.01) more positive than the evaluation of the “pathology examples” (2.6 ± 1.4), which were evaluated the worst alongside the “quiz” (2.5 ± 1.2). The greatest approval within the future perspectives was received for “teaching ultrasound skills during studies” (1.3 ± 0.9) and the “future value of blended learning” (1.4 ± 0.7).

**Table 2:** Results of the evaluation of the course design regarding the main and sub-items. 7-level Likert answering format from 1 = very good/completely to 7 = very bad/not at all

Item	T2	
	**Mean**	**SD**
**Future perspective**	1.5	0.5
Ultrasound in Medical Degree (general)	1.3	0.9
Ultrasound in degree program (compulsory)	1.8	1.1
Blended learning	1.4	0.7
**Course Design**	2.0	0.6
Course registration	2.2	1.4
Training concept (general)	1.7	0.7
Course design	1.6	0.8
Time commitment	1.9	1.1
Achievement of learning objectives	1.8	0.8
Pathology examples	2.6	1.4
Quiz	2.5	1.2
DOPS	2.0	1.0
Proportion theory vs. practice	1.5	0.7
Study materials (general)	1.9	1.0
Fulfilment of Expectations	1.9	0.9
**Exam formats**	2.3	0.9
Quiz	2.5	1.2
DOPS-scenarios	2.0	1.0
**Teaching materials**	1.7	0.6
**Lecture notes**	2.1	0.8
Structure	2.5	1.5
Scope	2.2	1.2
Content	1.8	0.9
Font/image size	1.8	1.3
Number of images/clips	1.8	1.2
Design/Layout	1.6	0.9
**Videos**	1.4	0.5
Duration	1.3	0.6
Technology	1.5	0.7
Content	1.5	0.7
**Tutors**	1.7	0.9
Professional competency	1.5	0.8
Didactic competency	1.8	1.3

### Objective tests

**Fig. 3:**
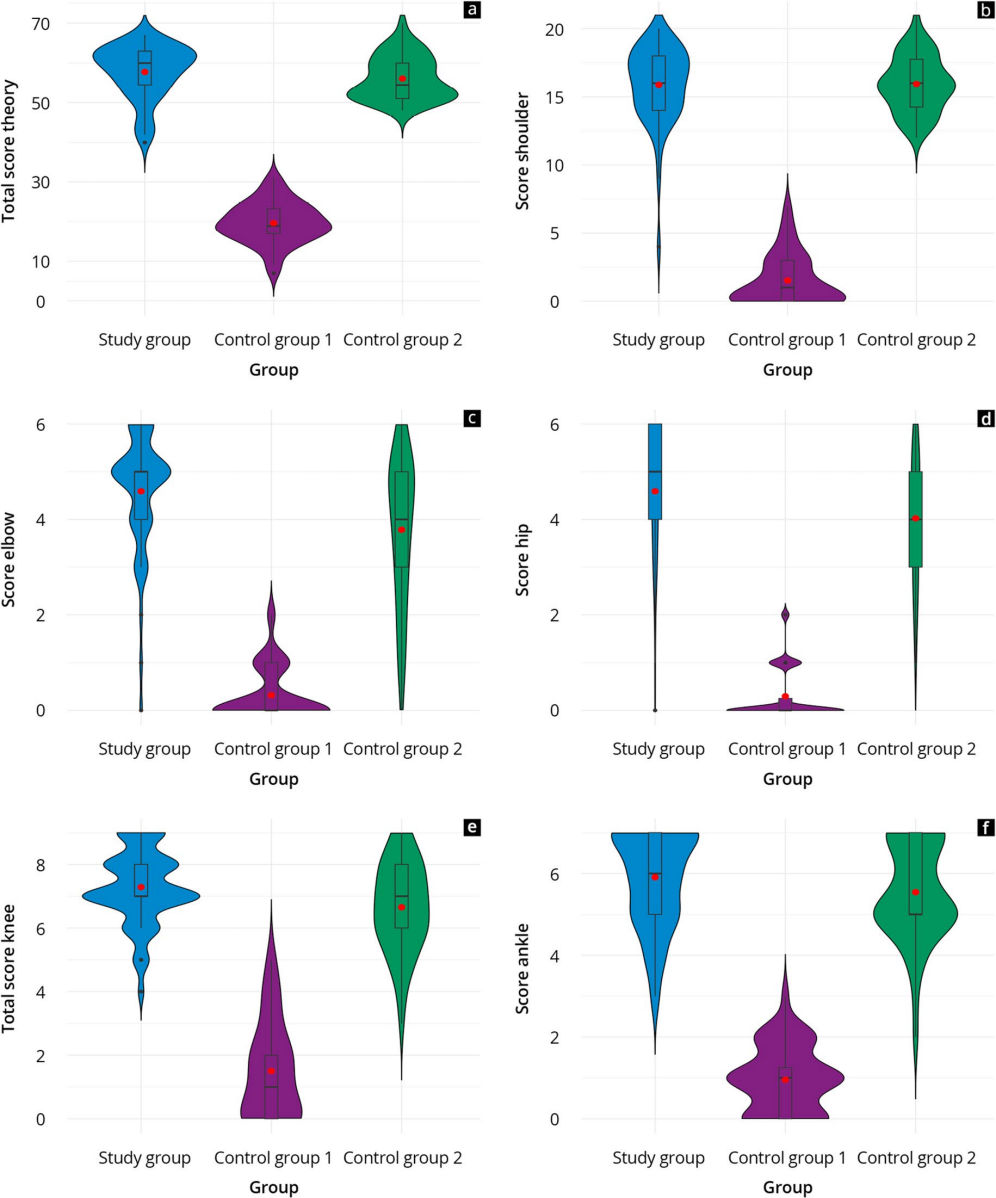
Results of the theory test of the study group (blue), control group 1 (purple) and control group 2 (green). **a** represents the overall score while the score per subcategory is given in **b**–**f**

**Fig. 4:**
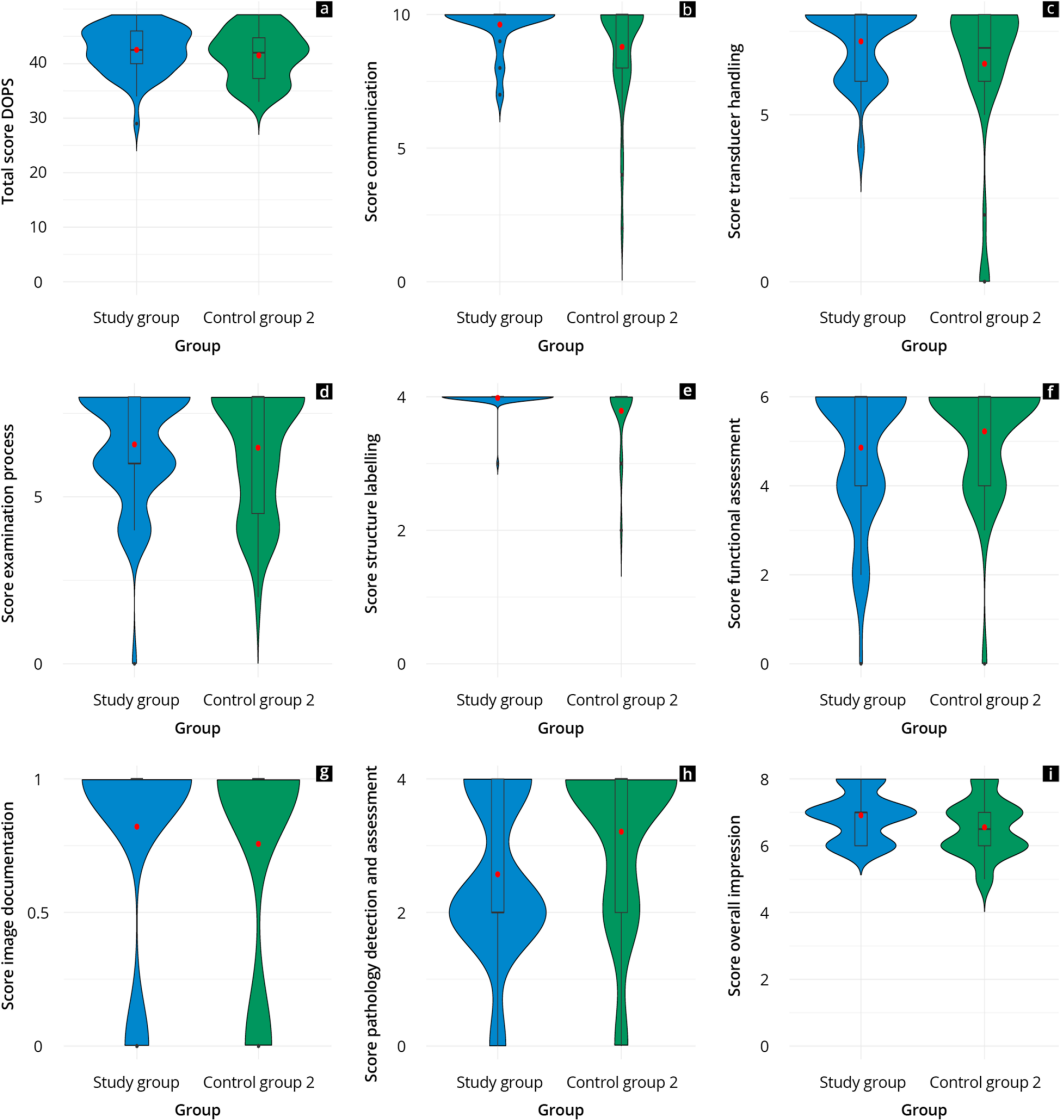
Results of the practical test of the study group (blue) and control group 2 (green) overall (**a**) and per subcategory (**b**-**i**)

The results of the theoretical and practical tests are presented in Figs. 3 and 4 and Supplement 6. The study group achieved average total values of ≥ 80% in the theory test, comparable to control group 2 (study: 58 ± 7 vs. control 2: 56 ± 5, *p* = 0.06). Both the study group and control group 2 had significantly higher scores in the overall value and all subcategories compared to control group 1 (*p* < 0.001). In both the study and control group 2, the categories “basics”, “knee”, and “feet” recorded the highest average grades. In the competency areas “elbow” (*p* = 0.004), “hips” (*p* = 0.03), and “knee” (*p* = 0.02), the study group achieved significantly better results than control group 2. For the rest, the two cohorts yielded comparable scores.

The study group achieved average overall values of ≥ 86% in the practical tests comparable to the performance of control group 2 (study: 43 ± 4 vs. control 2: 42 ± 5, *p* = 0.28). The study group performed better than control group 2 in the competencies “communication” and “overall impression” (*p* = 0.04), whereas “pathology detection/assessment” (*p* = 0.01) was significantly higher in control group 2. Both groups achieved the highest scores (≥ 95%) in the “labelling” competency.

### Influencing factors

The possible influencing factors of the performance in the practical test for the multivariable linear regression analysis of the results of the theoretical and practical tests of the studies and control group 2 were defined from the data and comprised “age”, “sex”, “group membership”, and “number of ultrasound examinations carried out”. Of these, the “number of ultrasound examinations carried out” significantly influenced the results of the practical test (β = 0.01, p = 0.048). No significant influence could be demonstrated for defined factors on the results of the theory test.

## Discussion

The blended-learning MSUS-specific training concept presented in this study, adapted from a postgraduate model, resulted in significant improvements in both subjective and objective competencies among the participating undergraduates. The theoretical and practical skills assessed at the end of the course were comparable to those of postgraduate medical doctors who had completed a similar curriculum. Furthermore, students provided positive evaluations of the training concept, supporting its future integration into medical degree programs. This study's novel approach to adapting and validating a physician-targeted training program for students highlights its potential to enhance MSUS education in undergraduate medical training, a topic not sufficiently explored in current literature.

### Subjective gain in competencies

Self-assessment of competencies is an important evaluation tool for many ultrasound training formats, allowing for the estimation of perceived competency gaps and promoting self-reflection [54]. It has been widely used in various MSUS-specific training concepts for both doctors [30, 33] and students [37, 38, 60]. Similar to previous studies [27], participants rated their skills significantly higher at the end of the training course, indicating a successful subjective increase in skills with this course design.

### Objective gain in competencies and influencing factors

Determining the acquisition or gain of objective competencies is crucial for estimating the effectiveness of a training concept [54]. The theoretical MSUS-specific knowledge acquired was assessed by multiple-choice questionnaires [27, 35, 40, 61] and free-text tasks, i.e. differentiating normal and pathological ultrasound appearances from previously saved images [40]. Standard cross-sections were shown to measure competencies regarding “normal findings” [47]. Theoretical competencies regarding “normal findings” were comparable to the results of previous studies [35, 40, 61], though examinations on previous studies did not assess all joint regions. Practical skills were assessed using Objective Structured Clinical Evaluation (OSCE) exams [35, 61] or Objective Structured Practical Examination (OSPE) [38]. The use of a modified OSAUS scale in the context of a DOPS examination should create a stronger correlation to a clinical setting, which can only be partially achieved in OSCE examination formats [32, 54, 58, 59]. The larger number of tasks, cross-sections and areas of competencies in the DOPS compared to the examination formats used in preliminary studies [62] could offer the advantage of creating a more differentiated proof of competency in subsequent clinical implementation. The particularly high scores in “fundamentals” could be explained by the fact that many students may have already taken an ultrasound course in another subject area (e.g., abdomen) during their studies. Participation in these could have enhanced visual-spatial ability and anatomical knowledge [62]. The higher values in the “knee” competency area compared to the “shoulder” align with previous studies [38] and can be attributed to the fewer tested sections of the knee and its less complex anatomy. In addition the shoulder is usually considered more complex compared to the knee by beginners because its sonographic assessment largely depends on its spatial position.

Practical MSUS skills, especially basic examination skills, have been shown to improve after both undergraduate [35, 40] and post-graduate courses [32]. This study reproduces these findings across more modules and joint regions compared to previous studies [35, 40, 50]. High values in "transducer handling" and "labelling" may result from participants’ prior ultrasound course experience and recent anatomy training [50]. The poorer results in “pathologies” should prompt more training, possibly through an advanced course, and extending assessment by written examination [54].

Overall, the level of practical competency achieved indicates successful implementation and teaching of MSUS-specific skills. In the future, other examination formats such as the Bright-mode Quality Ultrasound Imaging Evaluation Technique (B-QUIET) could be used to evaluate the images and observations recorded in clinical settings to document long-term learning success [63].

The two control groups allow for a better analysis of the acquisition of theoretical and practical skills. The similar high performance of both the study group and the medical control group 2, which was significantly higher than that of the untrained control group 1, suggest a significant gain in competencies even though the students were less experienced. The better results of the study group in the “communication” and “overall impression” competence areas may be due to OSCE and DOPS being integrated as examination formats elsewhere in undergraduate medicine recently, with students thus more aware of the general process and success requirements of these formats. The greater clinical experience of the doctors from control group 2 as well as the larger number of general examinations performed before the study could explain the better results of this group regarding “pathologies”. In addition, the certified medical course (control group 2) had a stronger focus on teaching pathology, which was not implemented in the transferred course format for students where the focus is on teaching basic examination skills.

### Course design

Other medical departments have previously successfully transferred a certified medical training concept to undergraduate teaching [64, 65]. These transferred concepts tend to include undergraduates who have completed at least the preclinical phase to ensure they have the necessary anatomical background to facilitate learning ultrasound [50]. Previous MSUS training concepts for students have typically focussed only on isolated joint regions such as the shoulder or knee [38, 61], or hand and wrist [40], or else the MSUS teaching forms a small part of a broader curriculum [37]. The teaching concept presented herein is based on a previous DEGUM concept [24, 33] with a total of 5 joint regions and thus a more comprehensive design than that of previous studies. The time frame of the training concepts for both user groups was implemented almost identically, with the students focussing on a stronger digital preparation phase and the ‘basics’ as well as sonoanatomy compared to the postgraduates.

The hand was intentionally excluded from the curriculum, as incorporating it would have expanded the course beyond its intended scope. This decision was made to maintain focus on core anatomical regions and ensure a balanced, manageable workload for participants, optimizing both the depth of learning and practical application within the available time. In our curriculum, the linear transducer was favoured and the convex transducer was used in exceptional cases to scan the hip. As in the published preliminary work on MSUS [27, 35, 38, 61] and other medical disciplines [64–67], the design was evaluated very positively by the participants overall and implementation in undergraduate tuition was desired, thus reflecting the recommendations of professional societies [21, 22].

The participants’ high satisfaction with the time commitment and balance of theory and practical sessions corresponds to evaluations from certified medical MSUS courses [33]. Designs spanning several weeks [68] could also be considered as a possible alternative in the future.

In postgraduate ultrasound training, blended learning and the use of digital teaching media and methods have recently been scrutinised in critical studies [69]. Blended learning, which has been increasingly implemented in radiological training [70], [27], was positively evaluated by students, particularly for its value in future tuition. In contrast to the MSUS training for doctors designed by DEGUM [24, 33] the digital preparation time using e-learning/online video courses was consciously planned and included as tuition in the study design, since as “digital natives”, the students use these digital teaching formats often, and the effectiveness of independent video-based training has already been shown in various preliminary studies [46, 71, 72]. In particular, the utilisation of mnemonic and metaphorical videos in MSUS has the potential to facilitate the comprehension of intricate anatomical concepts and enhance the retention of essential techniques by rendering them more accessible and memorable [46].Interaction on the online platform could be increased by using a digital forum in the future, and integrating digital teaching atlases may enhance the value of lecture notes [73].

Peer tutors may allow for longer training sessions spread throughout undergraduate tuition, but this strategy would require thorough implementation through ongoing training to ensure quality comparable to medical lecturers [35]. Even in this design, the inclusion of peer tutors reduced the group sizes from 6–14 students per teacher in preliminary studies [35] to 4–5 students as recommended by professional societies [21, 22]. Peer tutoring has already been implemented in other disciplines such as abdominal and cardiac sonography [65, 66].

In addition to reward systems and mentoring programmes, innovative teaching programmes such as the one in this study can generate enthusiasm among students and mitigate challenges in recruiting new colleagues to specialist medical disciplines. The positive evaluation of the MSUS teaching programme suggests the participants might have developed an ongoing interest in the specialties covered, such as orthopaedics or rheumatology. Indeed, the capacity for specific training to promote specialties has already been observed in the teaching of other clinical-practical (ultrasound) skills [67, 74, 75].

Another recent innovation enhancing musculoskeletal ultrasound training for beginners is the integration of artificial intelligence (AI) into ultrasound machines. AI provides real-time guidance, offering medical students and beginners feedback with a percentage score for longitudinal and transverse scans, ensuring accurate insonation of anatomical structures in both axes. This combination of physical and digital tutors is rapidly becoming the new standard in ultrasound education, significantly improving learning outcomes and precision [76].

### Limitations

The allocation of participants to the study and control groups was not randomized, and no pretesting of objective theoretical and practical skills was conducted. The small sample size limited subgroup analysis of the various six DOPS tests. The lack of follow-up testing precludes statements about long-term learning success In light of the potential limitations of our sampling method, we acknowledge that the findings of this study may not be generalisable to all undergraduate students. This is due to the fact that the sample may have been skewed towards highly motivated individuals with a pre-existing interest in ultrasound education. Tutor quality varied among the study and control groups. While standardized images and expert-recommended evaluation scales were used, final validation of the exam formats is pending. Practical tests were conducted on healthy volunteers, and pathologies were only tested in the DOPS.

## Conclusion

Despite its limitations, this study demonstrates that an MSUS-specific training curriculum designed for doctors could be successfully delivered to undergraduate students to achieve significant competency gains. The high satisfaction with the design supports its potential for integration into (extra-)curricular teaching at undergraduate level.

## Supplementary Information

Supplementary Material 1.

Supplementary Material 2.

Supplementary Material 3.

Supplementary Material 4.

Supplementary Material 5.

Supplementary Material 6.

## Data Availability

Data cannot be shared publicly because of institutional and national data policy restrictions imposed by the Ethics committee since the data contain potentially identifying study participants’ information. Data are available upon request from the Johannes Gutenberg University Mainz Medical Center (contact via weimer@uni-mainz.de) for researchers who meet the criteria for access to confidential data (please provide the manuscript title with your enquiry).

## Abbreviations

MSUS: Musculoskeletal ultrasound
DEGUM: German Society for Ultrasound in Medicine
DOPS: Direct Observation of Practical Skills Test
EULAR: European League Against Rheumatism
PANLAR: Pan-American League of Associations for Rheumatology
NKLM: National Competency-Based Learning Objectives Catalogue of Medicine
PT: Participants
DOPS: Direct Observation of Procedural Skills
OSCE: Objective Structured Clinical Evaluation
OSPE: Objective Structured Practical Examination
OSAUS: Objective Structured Assessment of Ultrasound Skills
AI: Artificial Intelligence
