# Supplementary material for: Undergraduate musculoskeletal ultrasound training based on current national guidelines—a prospective controlled study on transferability
Source: BMC Med Educ. 2024 Oct 23;24:1193. doi: 10.1186/s12909-024-06203-6 (PMC11515732; doi:10.1186/s12909-024-06203-6)
Supplement: Supplementary file 1 — Supplementary Material 1. [file 12909_2024_6203_MOESM1_ESM.pdf]

Supplement 1 Timetable of MSUS Curriculum

MSUS Course Students Online-Module

Duration	Content Online-Module	Lectures (45min)
15 min	Introduction	1/3
15 min	Basics of Underlying Physics and Technology(M1)	1/3
15 min	Knobology and Tissues (M1)	1/3
15 min	Phenomena and Artefacts (M1)	1/3
15 min	Shoulder Ultrasound (M2)	1/3
15 min	Elbow Ultrasound(M3)	1/3
15 min	Hip Ultrasound (M4)	1/3
15 min	Knee Ultrasound (M5)	1/3
15 min	Ankle Ultrasound (M6)	1/3
180 min	Self-study: Script and Sketching (M1-6)	4

MSUS Course Students Day 1

Time	Content Day 1	Lectures (45min)
09:00-09:15	Welcome Speech and Presentation of Programme<br>Feedback Questions	1/3
09:15-09:30	Introduction to devices + Knobology (M1)	1/3
09:30 – 09:45	Live-Demo Shoulder (M2)	1/3
09:45-11:15	Training Shoulder including Case Reports (M2)	2
11:15-11:30	Break	
11: 30-11:45	Live-Demo Elbow (M3)	1/3
11:45-13:15	Training Elbow including Case Reports (M3)	2
13:15-14:00	Break	
14:00-14:15	Live-Demo Hip (M4)	1/3
14:15-15:15	Training Hip including Case reports (M4)	1 + 1/3
15:15-15:30	Break	
15:30-15:45	Live-Demo Knee (M5)	1/3
15:45- 17:15	Training Knee including Case Reports (M5)	2
17:15-18:00	Pathology Quiz	1

MSUS Course Students Day 2

Time	Content Day 2	Lectures (45min)
09:00-09:15	Welcome Speech and Presentation of Programme	1/3
09:15-09:30	Live-Demo Ankle	1/3
09:30 – 11:00	Training Ankle including Case Reports (M6)	2
11:00-11:15	Break	
11: 15-12:45	Repetition of Examination Processes	2
12:45-13:30	Break	
13:30-14:00	Theoretical Assessment	2/3
14:00-15:00	Practical Assessment	1 + 1/3
15:15-15:30	Evaluation, Closing Remarks and Certificates	1/3

Theory: Digital= 3 units + 4 units Face-to-Face=4,66666 units → Total: 11,66 units

Training: 12,66666

Total: approx. 24 units of 45 min
