# Supplementary material for: Undergraduate musculoskeletal ultrasound training based on current national guidelines—a prospective controlled study on transferability
Source: BMC Med Educ. 2024 Oct 23;24:1193. doi: 10.1186/s12909-024-06203-6 (PMC11515732; doi:10.1186/s12909-024-06203-6)
Supplement: Supplementary file 2 — Supplementary Material 2. [file 12909_2024_6203_MOESM2_ESM.pdf]

Sample question 1

Which **structures and artefacts** are labelled with the arrows?

1. Artefact:
2. Artefact:
3. Artefact:

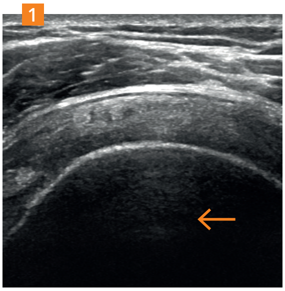

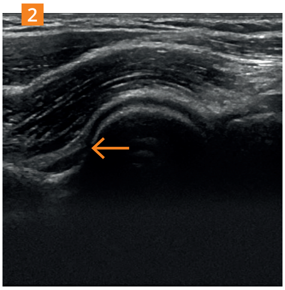

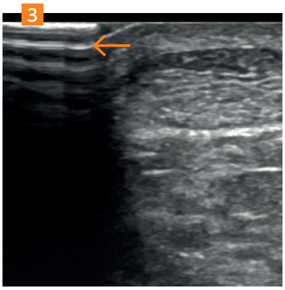

Sample question 2

Which **image modes** are shown in the images?

1. Image mode:
2. Image mode:

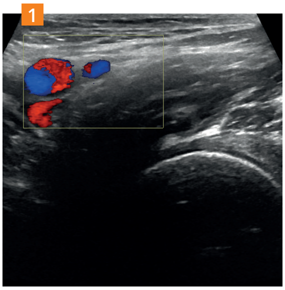

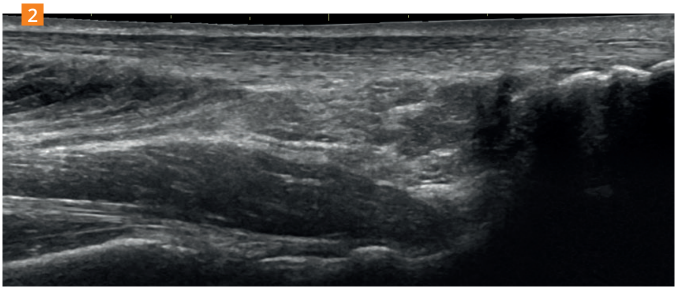

Sample question 3

Which **tissues** are labelled in the images?

1. Tissue:
2. Tissue:
3. Tissue:
4. Tissue:

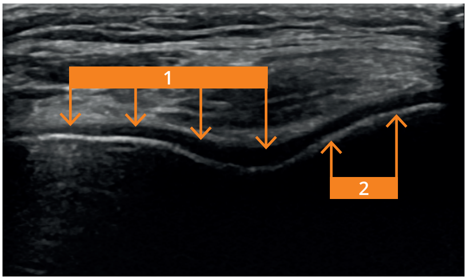

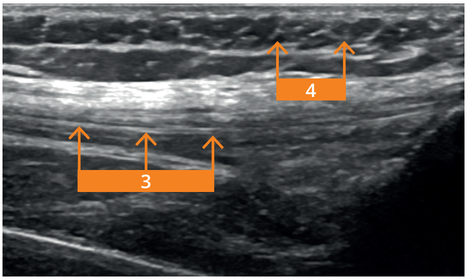

Sample question 4

· Complete the following sentence: The higher the frequency, the

____

the depth of penetration.

· Complete the following sentence: The lower the frequency, the

____

the depth of penetration.

Sample question 5

· Complete the following sentence: The correct sonomorphological term for "dark" areas in an image is:

____

· Complete the following sentence: The correct sonomorphological term for "light" areas in an image is:

____

Sample question 6

What **transducers** are shown in the images?

1:
2:
3:

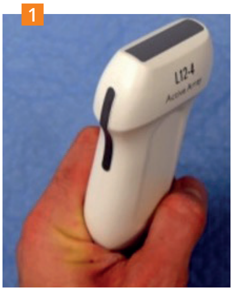

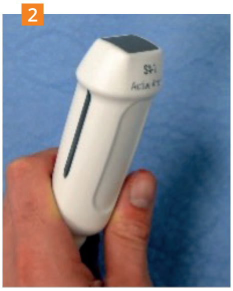

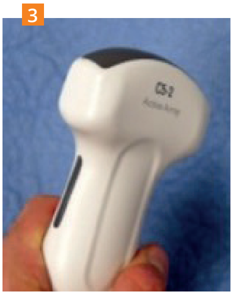

Sample question 7

Which **structures** are labelled in the sonographic image? Please state the kind of corss sectional image and describe the image.

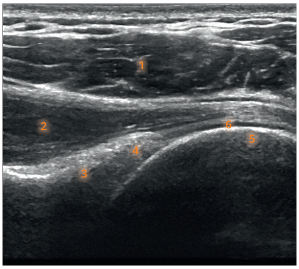
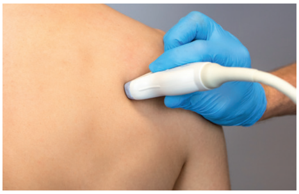

1: 2: 3:
4: 5: 6:

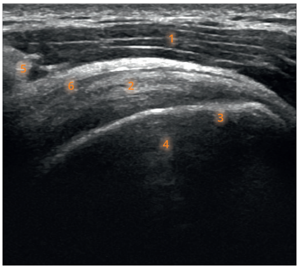
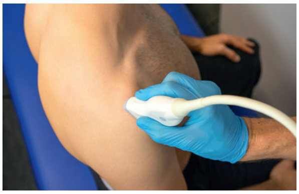

1: 2: 3:
4: 5: 6:

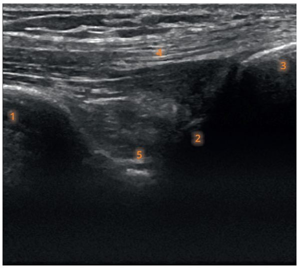
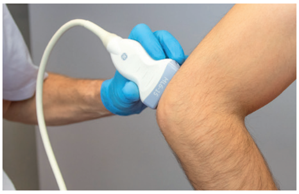

1: 2: 3:
4: 5:

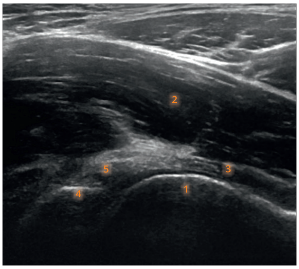
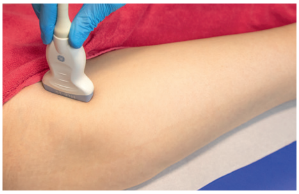

1: 2: 3:
4: 5:

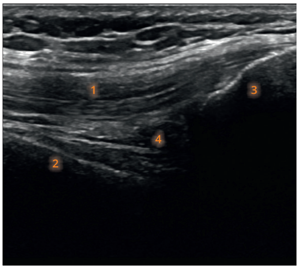
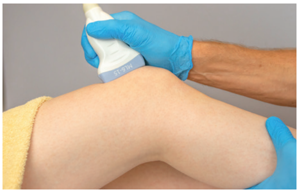

1: 2:
3: 4:

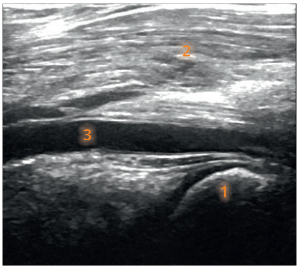
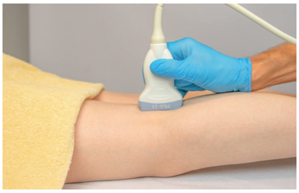

1: 2: 3:

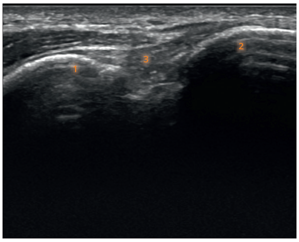
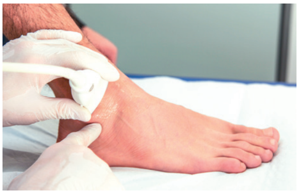

1: 2: 3:

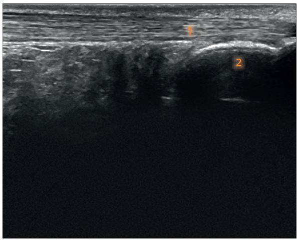
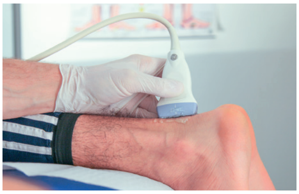

1: 2:
