# Supplementary material for: Undergraduate musculoskeletal ultrasound training based on current national guidelines—a prospective controlled study on transferability
Source: BMC Med Educ. 2024 Oct 23;24:1193. doi: 10.1186/s12909-024-06203-6 (PMC11515732; doi:10.1186/s12909-024-06203-6)
Supplement: Supplementary file 3 — Supplementary Material 3. [file 12909_2024_6203_MOESM3_ESM.pdf]

Supplement 3 Example sheet of the MSUS DOPS

DOPS No. ________: Topic _______ – evaluation form

Code participant:_________ examiner:___________

Task 1: Guide the patient appropriately through the examination. Name possible **indications / questions**.

**Patient management / Communication/ Indications** (max. 10 Credits)			
**Establishing a relationship**<br>Greeting, introducing yourself ,asking for name; asking about the patient's feeling	2	1	0
**Patient preparation**<br>Naming the occasion; asks about previous experiences; name the procedure and obtaining consent; indicates sufficient undressing; warning of sonic gel and light pressure	2	1	0
**Communication during the examination**<br>Empathic communication; pays attention to non-verbal and para-verbal communication; explains own actions and need for co-operation; gives clear instructions and understandable information	2	1	0
**Request for 'patient positioning' [...]**	2	1	0
Names **examination indications and questions**	2	1	0

Task 2:
Set the following **ultrasound sectional planes**: [...] and show the **structures / landmarks**: [...].

**Transducer-handling** (max. 8 Credits)	
**Orientation**	
Correct or immediately self-checked based on image movement / by uncoupling	2
Corrected after initial difficulties / after prompting	1
Correct orientation only found with manual help	0
**Positioning**	
Correct or immediately transferred from another section	2
Corrected after initial difficulties / after prompting	1
Correct orientation position only found with manual help	0
**Coupling / Transducer position**	
Transducer is coupled well with sufficient gel, continuous good pressure, good posture	2
Corrected after initial difficulties / after prompting	1
No pressure and/or no gel + discontinuous pressure and/or transducer uncoupled	0
**Adequate enlargement / image optimisation / device operation**	
Independent & adequate adjustment with appropriate image quality (gain, penetration depth, frequency, focus) even during examination, left hand on the device	2
Corrects after initial difficulties / after prompting, forgets optimisation during examination, does not keep left hand on the device	1
No adequate image setting despite request	0

**Screening** (max. 8 Credits)		
· Ultrasound section plane correctly set, appropriate tempo<br>· Ultrasound section plane set correctly, appropriate tempo	4	4
· Ultrasound section plane, inappropriate tempo / verbal help<br>· Ultrasound section plane, inappropriate tempo / verbal help	2	2
· Ultrasound section plane not set or only set with manual help<br>· Ultrasound section plane not set or only set with manual help	0	0

**Image explanation:** Correctly **pointing out and naming the structures** (max. 4 Credits)								
Structure 1	1 □	Structure 2	1 □	Structure 3	1 □	Structure 4	1 □	(…)

Example DOPS MSUS Weimer, Schneider, Berthold et. al. 2024

Task 3+4: Preise perform a **functional examination** (including side-by-side comparison) **of** [...] and **save** an **image / clip.**

**Measurement** (max. 6 Credits)		
· Left side correctly adjusted and functional test performed<br>· Right side correctly adjusted and functional examination performed	3	3
· Left side inaccurately adjusted and / or functional examination only performed with verbal help<br>· Right side inaccurately adjusted and / or functional examination only performed with verbal help	1	1
· Left Side adjustment and / or functional examination only performed with manual help<br>· Right Side adjustment and / or functional examination only performed with manual help	0	0
**Image documentation:** correct saving oft he image(max. 1 Credit)	1	0

Task 5: Interpret the submitted **examination report** after QR-code-scan and explain the **possible further procedure** (max. 4 Credits)

Correct pathology identification without hints	2
No correct pathology identification	0
Correct naming/suggestion of further diagnostic/therapeutic procedure	2
Incorrect naming/suggestion of further diagnostic/therapeutic procedure	0

Overall impression: I rate the overall impression with (max. 8 Credits – please circle)

1 – 2 – 3 – 4 – 5 – 6 – 7 – 8

Total Score: __/ 49
