# Supplementary material for: Undergraduate musculoskeletal ultrasound training based on current national guidelines—a prospective controlled study on transferability
Source: BMC Med Educ. 2024 Oct 23;24:1193. doi: 10.1186/s12909-024-06203-6 (PMC11515732; doi:10.1186/s12909-024-06203-6)
Supplement: Supplementary file 4 — Supplementary Material 4. [file 12909_2024_6203_MOESM4_ESM.pdf]

Supplement 4 Baseline characteristics of sample per group.

Item	Study group (study) N=56	Control group 1 (C1) N=44	p-value study vs. K1	Control group 2 (C2) N=46	p-value study vs. C2
Registered for course	90	60	/	55	/
Attended course	66	58	/	52	/
Completion and participation in study	56	44	/	46	/
**Age (mean±SD)**	25±4.0	27 ±3.3	<0.001	37 ±8.7	<0.001
**Sex**					
Male	31 (55%)	13 (30%)	0.01	29 (63%)	0.6
Female	25 (45%)	31 (70%)		17 (37%)	
**Qualification**					
Student	56	42	0.1	/	/
Students in practical year	0	2		/	
Resident	/	/		27	
Specialist	/	/		15	
Consultant	/	/		4	
**Discipline**					
Orthopaedics Trauma surgery	/	/	/	35	
Nephrology Rheumatology Internal Medicine	/	/	/	5	
Radiology	/	/	/	2	
Family Medicine Occupational Medicine	/	/	/	2	
Others	/	/	/	2	
**Ultrasound Examinations (irrespective of Organ system or region)**					
Observed	32±40	15 ±19	0.005	174±230	<0.0001
Performed	13±16	8.8 ± 13	0.11	158 ± 1779	<0.0001
