# Supplementary material for: Undergraduate musculoskeletal ultrasound training based on current national guidelines—a prospective controlled study on transferability
Source: BMC Med Educ. 2024 Oct 23;24:1193. doi: 10.1186/s12909-024-06203-6 (PMC11515732; doi:10.1186/s12909-024-06203-6)
Supplement: Supplementary file 5 — Supplementary Material 5. [file 12909_2024_6203_MOESM5_ESM.pdf]

Supplement 5 Participants' subjective development of competency rated with Likert answering formats (1=very high; 7=very low).

Item	T1		T2		p-value
	Mean	SD	Mean	SD	
**Ultrasound Skills (total)**	**4.8**	**1.1**	**2.1**	**0.7**	**<0.001**
**General ultrasound skills**	**4.2**	**1.0**	**2.1**	**0.6**	**<0.001**
Theoretical knowledge	3.8	1.6	2.3	0.7	<0.001
Equipment use	3.3	1.6	2.1	0.8	<0.001
Transducer handling	2.9	1.4	1.8	0.7	<0.001
Spatial orientation	5.4	1.4	2.0	0.9	<0.001
Sono anatomical assignment	4.4	1.5	2.1	0.8	<0.001
Soft-Tissue visualization	4.7	1.7	1.9	0.9	<0.001
Soft-Tissue assessment	5.0	1.6	2.1	0.8	<0.001
Patient guidance	3.6	1.7	2.0	0.8	<0.001
**MSUS-specific Skills**	**5.3**	**1.7**	**2.2**	**0.7**	**<0.001**
Shoulder	5.3	1.9	2.1	0.9	<0.001
Elbow	5.6	1.6	2.0	0.8	<0.001
Hip	5.3	1.9	2.3	0.9	<0.001
Knee	5.3	1.9	2.0	0.8	<0.001
Ankle	5.6	1.7	2.1	1.0	<0.001
