# Supplementary material for: Undergraduate musculoskeletal ultrasound training based on current national guidelines—a prospective controlled study on transferability
Source: BMC Med Educ. 2024 Oct 23;24:1193. doi: 10.1186/s12909-024-06203-6 (PMC11515732; doi:10.1186/s12909-024-06203-6)
Supplement: Supplementary file 6 — Supplementary Material 6. [file 12909_2024_6203_MOESM6_ESM.pdf]

Supplement 6 Theoretical and practical test results of the studies and control groups given in percent.

Item	Study group (study)	Control group 1 (C1)	study vs. C1	Control group 2 (C2)	study vs. C2
	**Mean %±SD**	**Mean %±SD**	**p-value**	**Mean %±SD**	**p-value**
**Theory test**	**80 ± 9**	**27 ± 7**	**<0.001**	**78 ± 8**	**0.06**
Basics	84 ± 10	66 ± 10	<0.001	88 ± 5	0.32
Shoulder	76 ± 13	7 ± 9	<0.001	76 ± 10	0.6
Elbow	77 ± 20	5 ± 10	<0.001	63 ± 25	0.004
Hip	77 ± 23	5 ± 8	<0.001	67 ± 23	0.03
Knee	81 ± 12	17 ± 17	<0.001	74 ± 16	0.02
Ankle	86 ± 17	14 ± 11	<0.001	79 ± 20	0.06
**Practical test**	**87 ± 8**			**85 ± 2**	**0.28**
Communication	96 ± 8			88 ± 19	0.01
Transducer handling	90 ± 14			81 ± 28	0.25
Examination process	83 ± 21			81 ± 23	0.89
Structure labelling	100 ± 3			95 ± 13	0.01
Functional assessment	82 ± 25			85 ± 22	0.11
Image documentation	80 ± 40			80 ± 50	0.67
Pathology detection and assessment	65 ± 30			80 ± 33	0.01
Overall impression	86 ± 9			83 ± 10	0.04
